# Lipid Alterations and Metabolism Disturbances in Selected Inflammatory Skin Diseases

**DOI:** 10.3390/ijms24087053

**Published:** 2023-04-11

**Authors:** Julia Nowowiejska, Anna Baran, Iwona Flisiak

**Affiliations:** Department of Dermatology and Venereology, Medical University of Bialystok, Zurawia 14 St., 15-540 Bialystok, Poland

**Keywords:** lipidomics, lipids, psoriasis, atopic dermatitis, lichen planus, hidradenitis suppurativa, acne vulgaris, rosacea, seborrheic dermatitis

## Abstract

Lipidomics is a term used to define the field that analyzes the structure, functions, and interactions of lipids. Inflammatory dermatoses and lipid disturbances are interrelated, especially due to chronic inflammatory conditions. This review discusses lipidomics in selected inflammatory skin diseases: psoriasis, lichen planus, and atopic dermatitis, as well as the less commonly mentioned hidradenitis suppurativa, rosacea, and acne vulgaris. Lipid homeostasis disorders are common; they are especially well-documented in psoriasis, lichen planus, and atopic dermatitis. Future studies are required for better insight into this issue, particularly on the skin lipidome. Understanding lipidomics, in particular skin diseases, increases our knowledge about their pathogenesis, and may become useful in adjusting tailored management for each patient as well establishing prognosis. Noteworthily, it seems advisable to alert doctors to the need to analyze lipid parameters and the complications of abnormal lipid metabolism in dermatological patients, which could decrease their comorbidities and improve the life quality and health condition of dermatological patients.

## 1. Introduction

**Figure 1:**
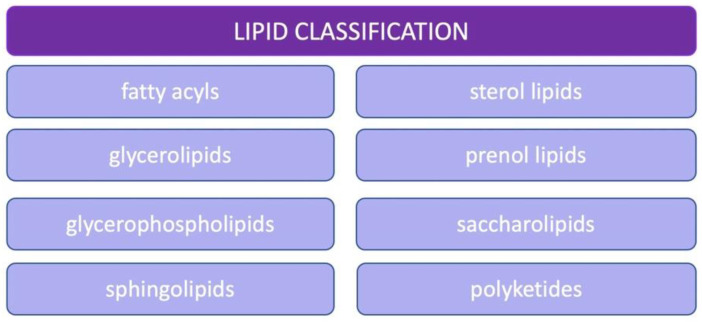
Updated classification of lipids.

Lipidomics is a distinct branch of metabolomics; it refers to the field that analyzes the structure, functions, and interactions of lipids [1,2]. Probably the most updated classification of lipids is the one presented by the LIPID MAPS consortium, which has divided lipid molecules into eight groups: fatty acyls, glycerolipids, glycerophospholipids, sphingolipids, sterol lipids, prenol lipids, saccharolipids, and polyketides (Figure 1) [1,3].

The matter of lipids is complex as the composition and distribution of different kinds of lipids described in distinct organism species, organs, tissues, cells, and even cellular organelles is highly variable [1]. Thus, lipids have a wide variety of characteristics and functions. First of all, they are components of cellular membranes, which may determine their properties and are engaged in cellular transport, signal transduction, cell proliferation, and apoptosis [1,2]. Moreover, they are involved in energy management and immune response [1,2].

The intercellular lipids in the epidermis are important for the maintenance of moisture and elasticity, which provides a proper epidermal barrier [4]. The most prominent role in skin metabolism is played by the eicosanoids, endocannabinoids, and sphingolipids, which are present in the epidermis and dermis [5]. Local disturbances in lipid quantity and composition have been documented as important factors in several skin diseases [4]. However, even in healthy subjects there is a degree of variety between lipid components in the skin [4].

Apart from skin lipids, lipid compounds in the blood play essential functions in healthy subjects, and disturbances are involved in different dermatological diseases. Conditions associated with abnormal blood lipid composition (e.g., dyslipidemia, diabetes mellitus, atherosclerosis) may influence skin lesion severity, and vice versa; certain dermatoses have been proven to have an impact on lipid disturbances [6].

Lipidomics has drawn much attention in different fields, including medicine. Research concerning lipid homeostasis and aberrations in human body has begun in every area of medicine, and has led to interesting outcomes that could be further applied in daily medical practice.

Lipid aberrations are frequent in several inflammatory skin diseases, which leads to health implications for such patients, affecting both the choice of appropriate treatment and its outcomes. Noteworthily, such aberrations have been described in the blood and, in certain dermatoses, in the skin. The analysis of epidermal lipidome has been suggested as a tool for predicting of the progression of inflammatory skin diseases [4], which is a concept worthy of attention. Unfortunately, for many diseases these data are limited.

The present review discusses lipidomic aspects in several inflammatory dermatoses, in particular two of the most common chronic skin diseases, psoriasis and atopic dermatitis, as well as several others: lichen planus, hidradenitis suppurativa, acne vulgaris, rosacea, and seborrheic dermatitis, and aims to provide the current state of knowledge and insight into their frequency, complications, management, and possible applications in clinical practice.

## 2. Psoriasis

Psoriasis is one of the most common chronic and incurable dermatoses in daily dermatological practice, and is one of the main fields of research in dermatology. It affects about 125 million people worldwide [7]. Psoriasis pathogenesis is complex, and is not yet fully understood. It involves a combination of genetic factors, immunological disturbances, and environmental stimuli [8]. The most common type of psoriasis is plaque psoriasis, which clinically presents as erythematous scaly plaques in the area of the extensor surfaces of the limbs, lumbar–sacral area, and gluteal fold [9]. Treatment of psoriasis is long-lasting, time-consuming for the patient, and challenging for the doctor. Topical, as well as systemic agents may be used. Currently, the topical treatment of choice is calcipotriol with betamethasone. Among the classic systemic therapies, the following are used: methotrexate, acitretin, cyclosporin A, dimethyl fumarate, and apremilast. Moreover, there are biological drugs available in the form of antibodies against interleukins or their receptors, targeting TNFα, IL17, IL12/23, or IL23 [10,11].

At the same time, psoriasis is probably the dermatosis most closely associated with lipid aberrations, which is well-documented [12]. The first reports on the possible association of psoriasis with metabolic syndrome (MS) are from 2006 [13]. Subsequently, the number of studies on this matter has exploded. Psoriatic patients are known to have aberrations in lipid composition in both the blood and epidermis, and to have increased risk of MS, including dyslipidemia and obesity, accompanied by atherosclerosis or non-alcoholic fatty liver disease (NAFLD) [6].

In general, the following lipid abnormalities are observed in such patients’ blood: elevated concentration of total cholesterol, triglycerides, low-density lipoprotein (LDL), very-low-density lipoprotein (VLDL), and apolipoprotein B. On the contrary, high-density lipoprotein (HDL) concentrations are significantly decreased in psoriatics. In the case of apolipoprotein A, there is probably no difference between patients with psoriasis and controls [14]. As for phospholipids, research has shown that the total concentration of phospholipids, phosphatidylethanolamine, lecithin, linolenic acid (LA), and docosatetraenoic and docosapentaenoic acids are lower in psoriatics [15,16]. Plasma concentrations of lysoglycerophospholipids, such as lysophosphatidic acid and lysophosphatidylcholine, and phosphatidic acid have been reported to be significantly increased in psoriatic patients [2]. The data regarding palmitic and palmitoleic acids are less coherent; studies have shown either elevated or similar concentrations compared to non-psoriatic subjects [15]. As for concentrations of adipokines released by the adipose tissue, these are altered in psoriasis. Serum levels of leptin, chemerin, progranulin, resistin, and visfatin are increased, whereas adiponectin, vaspin, and omentin are decreased [6].

For lipids in the psoriatic epidermis, a decreased content of free fatty acids and an increased of cholesterol have been reported [15,17]. Among the different kinds of ceramides, their composition is abnormal in the area of psoriatic plaques [17]. The expression of adipokines in psoriatic skin compared to subjects without psoriasis is different as well, corresponding to what is observed in the blood. Leptin, progranulin, chemerin (in early phase lesions), resistin, visfatin, and serpin 1 are elevated, whereas adiponectin, vaspin, omentin, and chemerin (in chronic lesions) are decreased in psoriatic plaques [6]. Research has shown altered expression of several enzymes engaged in the lipid metabolism within psoriatic skin lesions. The expression of serin palmitoyltransferase and sphingomyelinase is decreased compared to unlesional skin [17,18]. In addition, there are reports of decreased activity of ceramide synthases and elongases due to inhibition by interferon γ [19].

Because the association between psoriasis and lipid disturbances is well-established, specific management recommendations have been made. According to joint AAD-NPF guidelines, dermatologists should inform patients about the increased risk of MS and cardiovascular incidents and refer them to their primary care provider or cardiologist for appropriate screening, such as measurement of height, weight, blood pressure, blood glucose, hemoglobin A1C, lipid levels, and abdominal circumference. Moreover, attention should be paid to potential lifestyle modifications [20].

## 3. Lichen Planus

Lichen planus (LP) is another chronic inflammatory skin disease; it affects about 1% of the population [21]. It has been suggested that LP may be genetically determined and an autoimmune disorder. Moreover, there are triggering factors that may lead to the appearance of the disease; these are infections with hepatitis viruses, intake of certain drugs, and stress [22]. LP may affect the skin, mucosa, nails, and scalp, whereas cutaneous LP is the most common manifestation [23]. The clinical picture of its classic type is characterized by the presence of multiple polygonal, purple, flat-topped, pruritic papules that can be located anywhere on the body, usually on the wrists, forearms, and legs [23]. The treatment may vary depending on the subtype and localization of the disease. Usually, the methods used are topical or oral glucocorticoids, phototherapy, or acitretin [24].

LP is probably the second-most common dermatosis that is that tightly associated with lipid disturbances. In recent years, there has been growing interest in this matter. The majority of available studies suggest an increased risk of MS in LP subjects. The estimated frequency is distributed from 6% to 77% [21]. There have been several studies thus far in which there was a higher prevalence (significantly or insignificantly) of MS in LP patients [25,26,27,28]. One of these studies, by Dreiher et al., investigated different components of MS in subjects with LP based on a large cohort; there was a statistically significant difference in the frequency of dyslipidemia between the groups, but not in the rest of the conditions (obesity, DM or arterial hypertension) [29]. On the subject of dyslipidemia, the majority of available studies show especially significantly increased occurrence of hypertriglyceridemia in LP patients [27,30,31,32]. Moreover, there is an apparent tendency of increased LDL and decreased HDL in this group [26,27,32]. One of the latest studies assessed other lipid particles as well. Patients had significantly higher concentrations of the following compounds compared to controls: total and free cholesterol, cholesteryl esters, LDL, and intermediate-density lipoproteins [33]. There were differences, however insignificant, between patients and the control group in the case of serum sphingomyelins, LA, polyunsaturated fatty acids (PUFA), and omega-6 fatty acids [33].

As for atherosclerosis, there are a few studies on LP subjects, all indicating an increased risk of this condition based on carotid intima–media thickness, a marker of subclinical atherosclerosis [27,31,32]. There are no reports on NAFLD in LP.

To the best of our knowledge, there is no research regarding the composition of lipids in the skin samples of patients with LP. This area may become a potential future research target.

LP research regarding MS is difficult to interpret due to the variety of LP involvement sites and subtypes. Obviously, the majority of papers concern cutaneous and oral LP. Even though LP has emerged as another dermatosis associated with lipid disturbances, there are no specific recommendations established regarding the management of such patients.

## 4. Atopic Dermatitis

After psoriasis, atopic dermatitis (AD) is one of the most common chronic skin diseases. The pathogenesis of AD is complex, including genetic background, immune disturbances, and modulating environmental factors [34]. The hallmark of AD is the dysfunction of the epidermal barrier, which results in loss of water and subsequent severe dryness, in turn leading to easier penetration of pathogens and allergens [34]. The clinical presentation is dependent on the age of the patient; in adults, there are erythematous and exfoliating lesions with lichenification on the flexor surfaces of limbs with accompanying severe pruritus [35,36]. Noteworthily, AD is a heterogenic disease, and there are significant racial differences in pathogenesis and clinical picture observed which should be taken into consideration [37]. The diagnosis is made based on the Hanifin and Rajka criteria [34]. The foundation of therapy is daily skin care, especially the application of emollients. Moreover, topical calcineurin inhibitors or steroids can be used. In more severe cases phototherapy, oral immunosuppressive agents and biological drugs are introduced [34].

However, AD mainly affects children and tends to resolve during growth [34]. There have been studies regarding lipidomics involving both adults and children, mostly on the former. Lipids are essential in AD subjects, as they are involved in the skin moisturizing and inflammatory processes [38].

Based on the available literature, a conflicting association between AD and MS is found; moreover, there is a large difference between studies in terms of the number and age of enrolled participants. Probably the largest available analysis to date was performed on 116,816 adults and children of mixed ethnicity with AD [39]. Taken together, these patients had significantly less frequent MS and DM in general and more frequently dyslipidemia, with no significant differences in obesity or arterial hypertension [39]. When analyzing only adults, patients present obesity significantly less frequently, and when taking into account only adults with moderate to severe skin lesions, they have more prevalent MS and its components in general: obesity, DM, dyslipidemia, arterial hypertension, and even heart disorders, which may point to the role of skin lesion severity in these associations [39]. Another large cross-sectional study on over 500,000 adult subjects including 13,822 patients with AD revealed that AD patients had significantly lower triglycerides and total cholesterol [40].

On the contrary, there have been several studies and reviews which claim otherwise, though these were performed on smaller samples or different ethnicities, which must be noted [36,41]. The postulated association between AD and MS may be explained by dysregulation of the immune system. As with psoriasis, adipose tissue secrets soluble adipokines, affecting inflammatory and immune processes, which in turn stimulate the production of pro-inflammatory cytokines, which sustain the chronic inflammatory condition and provide a higher predisposition to hypersensitivity reactions [36]. Among the particular components of MS, there are reports on a positive association between AD and central obesity in both children and adults [36,41,42,43]. Again, similar to psoriasis, the relationship seems bidirectional; AD subjects are predisposed to obesity, and obesity enhances inflammation in AD [36]. As for dyslipidemia, there is evidence of its increased frequency among AD patients. Several studies have reported on significantly increased serum concentrations of total cholesterol, LDL and triglycerides [36,44]. As for HDL, the data seem inconsistent; in some research its concentration has been reported to be decreased, while in others it has been reported to be increased [36]. The first case reflects the propensity of AD patients to dyslipidemia, whereas the second reflects a compensatory mechanism in the subjects [36]. In addition, there is research focused on the alterations of HDL composition in AD patients. For the HDL-associated proteins, the most common apolipoprotein has been revealed to be apo-AI; however in those patients apo-AII and SAA were significantly more frequent. ApoC-III and apoE in AD patients were significantly decreased. ApoC-III and apoE in AD patients were significantly decreased. There was no significant difference in apoC-II content in patients [45]. As for the HDL-associated lipids, AD patients had significantly lower cholesteryl esters, free cholesterol, phosphatidylethanolamine, and lysophosphatidylcholine than controls, as well as higher phosphatidylinositol. There were no significant differences in the content of HDL-associated phosphatidylcholine, triglycerides, and sphingomyelin [45]. Noteworthily, in the same study isolated eosinophils of healthy volunteers were pretreated with HDL taken from the control group without AD and AD patients. Depending on the concentration of lysophosphatidylcholine, enriched HDL suppressed eosinophil shape change and migration or reduced eosinophil shape change had no inhibitory effect on eosinophil migration [45].

Of note, a correlation has been identified between dyslipidemia and the severity of skin lesions using the SCORAD scale [36,44]. Moreover, scientists have revealed an association between abnormal lipid parameters and susceptibility to hypersensitization and risk of AD development [36,44]. Other interesting state-of-the-art information includes that the proinflammatory lipid index in the cord blood of newborns is associated with the risk of early AD onset [46].

With regard to the composition of eicosanoids in AD subjects’ blood, studies have shown a significant drop in lipoxin A_4_, leukotriene B_5_, docosahexaenoic acid (DHA), and maresin, as well as a slight drop in 5-hydroxy-eicosapentaenoic acid (5-HEPE), and eicosapentaenoic acid (EPA) concentrations. Reported LA concentrations differ among studies [47].

Data on other components of MS (arterial hypertension or hyperglycemia) in AD are somewhat incoherent [42]. Considering atherosclerosis, increased concentrations of molecular atherosclerotic markers have been detected in persons with AD. Those were, for instance fractalkine, CCL4, CCL17, CCL28, CXCL5, CXCL10, and hepatocyte growth factor (HGF). Elevated IL-20 has been found as well, and is known to promote hyperplasia of keratinocytes and atherosclerosis [36].

Lipid components have been analyzed in the skin of AD patients. Research has revealed that the total level of ceramides as well as their several subclasses (CERNH], CERNP], CEREOS], CEREOH], CEREOP]) and those with short-length chains (CERNS], CERNDS], CERAS]) were lower in the lesional AD skin compared to the skin of healthy subjects. The long-length chain ceramides, (CERNS], CERNDS], CERNH], CERAS], CERAH]), on the other hand, were higher in patients with AD [4,48]. There are reports that the alterations in the lengths of ceramides chains are correlated with improper lipid composition and dysfunction of the epidermal barrier, which later translates into AD skin lesion severity [4]. For instance, Ishikawa et al. proved that one particular short-chain CERs class is elevated in AD skin lesions, and is correlated with disruption of skin barrier [48]. On the other hand, Janssens et al. analyzed non-lesional skin in AD patients and confirmed that the length of CER chains matters. This reduced length was correlated with dysfunction of epidermal barrier expressed through transepidermal water loss. The authors concluded that the chain length of CERs is more relevant than the altered ratio of different CER subtypes in regard to lipid organization and barrier permeability. Moreover, the altered composition of CERs was associated with AD severity as expressed by SCORAD and level of natural moisturizing factor, though notably not with filaggrin genotype [35]. There was no difference in total CER concentration between non-lesional skin and skin of non-AD subjects [35]. This study indicates a possible future research path targeting CER chain length as a therapy grip point. Lipid compounds in the sweat of AD patients have been studied, which seems to be an excellent idea considering the non-invasive nature of this procedure. Studies have shown that AD influences sweat lipid composition and that there is an imbalance between distinct ceramide subclasses [38,49]. For instance, AD patients present increased concentrations of [NS] and [NdS] CERs and C18:1 sphingosine in their sweat [49].

Interestingly, alterations in the epidermal lipid composition have been found to differ between distinct body sites and to correlate with increased colonization by *Staphylococcus aureus* [50].

An important source of bias in the case of AD may be the fact that the majority of studies have been performed on adult patients or have not clearly defined the age of the participants; hence, we do not really have true information on how lipidomics presents in most patients, who in practice are typically children. Of note, atherosclerosis and dyslipidemia usually occur in older patients, which may affect the interpretation of the results. Moreover, it is important to pay attention to whether studies were performed on lesional or non-lesional skin samples.

Similar to LP, there are no guidelines regarding screening for metabolic disorders in this group at the moment.

## 5. Hidradenitis Suppurativa

Hidradenitis suppurativa (HS) is a rare yet important dermatosis, as it has a significant negative impact on the quality of life of patients [51]. The exact frequency of HS in the general population is unknown, as the condition is often underrecognized; however, it is estimated to be about 0.00033–4.10% [51]. The clinical presentation involves recurring nodules, abscesses, fistulae, and scars that appear in the intertriginous areas, such as the armpits, groins, submammary folds, and perianal area. Lesions are accompanied by pain [51]. While the pathogenesis of HS is not fully understood, the current data point to follicular hyperkeratosis as a primary finding, followed by plugging and dilation of the follicle, results in its rupture and inflammation [51]. The treatment of HS is very difficult, combining pharmacological and surgical approaches, including smoking cessation [5].

According to the literature, the updated estimated odds ratio (OR) of HS patients for MS development are 2.66 (95 CI: 1.90–3.72) [52]. First, there is a significant association between general obesity (expressed by BMI) and abdominal obesity (expressed by waist circumference) in HS patients [53,54,55]; moreover, there is an association between dyslipidemia, DM, and arterial hypertension [54,55], and there is evidence of an association between HS and NAFLD [55].

A study by Miller et al. on a large group of patients obtained meaningful results considering lipidomics in HS. For blood lipids, available studies suggest that HS might be associated with lipid disturbances. A significant association between HS and high triglyceride concentrations has been reported in a previously mentioned large study by Miller et al., along with low concentrations of HDL [53]. In newer research with a smaller group by Hernandez et al., a significantly lower HDL was present in patients compared to controls however, no significant differences were obtained for triglycerides or LDL concentrations, and surprisingly the controls without HS had significantly higher concentrations of total cholesterol than patients with HS [56]. Noteworthily, the same study showed that the atherogenic index of plasma is associated with the severity of HS, which could be a useful clue in clinical practice [56]. There was no significant difference in apolipoprotein A1 or B between patients and controls [56].

Considering skin lipidomics, a study by Penno et al. compared lipid contents between lesional skin and non-lesional skin in HS patients and non-HS subjects [5]. Most of the analyzed lipid mediators revealed no significant differences between HS and healthy skin. The only significantly increased lipids in the lesional skin were 5-LO-derived metabolites of omega-6 fatty acids. On the other hand, omega-3 fatty acids and docosahexaenoic acid were significantly decreased in HS lesions [5].

Considering the wide comorbidity of HS, there are a number of recommendations for management. It is advisable to refer HS patients for additional tests in search of cardiovascular comorbidities, especially in cases in which the patient presents alarming symptoms or smokes cigarettes [57].

## 6. Seborrhoeic Dermatitis

Seborrhoeic dermatitis (SD) occurs in about 1–3% of the population across all races and ages [58]. The clinical presentation is erythematous and scaly lesions located on the face, scalp, and chest [58]. Its pathogenesis involves lipid secretion from the sebaceous glands, which are subsequently broken down by *Malassezia* sp., leading to the release of free fatty acids. These trigger inflammation and pro-inflammatory cytokines stimulate the proliferation of keratinocytes, leading to disruption of the epidermal barrier and resulting in erythema and exfoliation [58]. Treatment involves topical anti-inflammatory agents (calcineurin inhibitors or glucocorticoids) and topical or oral antifungal drugs [59].

Considering blood lipids, Akbas et al. reported that patients with SD tend to have significantly higher total cholesterol concentration and LDL along with lower HDL [60]. The same authors suggested that the presence of MS, particularly dyslipidemia, may increase the risk of SD appearance and its severity [60]. They suggested that involvement of particular sites with SD lesions may be more predictive of MS, especially the scalp, eyebrows, and nasolabial folds [60]. Imamoglu et al. did not observe any significant differences between patients and controls except for lower HDL [61]. The consequence of decreased HDL in SD may be the downregulation of its antimicrobial properties, which subsequently leads to increased colonization with *Malassezia* and consequent inflammatory conditions [61].

There has been considerable research regarding skin lipidomics in patients with SD. In older studies from many years ago, patients with SD were discovered to have decreased squalene, free fatty acids, and wax esters, as well as elevated levels of cholesterol, triglycerides, and paraffins [62]. Similar results were obtained by Passi et al., who studied patients with SD who were HIV-negative; patients had significantly higher content of cholesterol within the skin lesions, a lower level of squalene, and lower total lipid capacity compared to controls without SD [63]. There were no significant differences in free fatty acids or triglycerides between the groups [63]. In another study, no significant changes were observed in lesional skin in SD subjects [64]. In the newest study by Suchonwanit et al., mean skin surface lipids were higher in SD than in healthy subjects, which was correlated with SD severity [65]. Possible alterations in lipid composition are probably present due to abnormal keratinization process in the lesional skin [63]. Increased sebum secretion, which is one of the pathogenic elements in SD, has been proven to dysregulate desquamation and lipid organization in the stratum corneum of the epidermis [65].

Up-to-date information on the pathogenesis of SD indicates that seborrhoea is not necessarily required for disease occurrence [58]; moreover, based on our review, the matter of lipidomics in SD seems insufficiently investigated to date, and more studies are needed in the future.

## 7. Rosacea

Rosacea is a chronic skin disease that occurs in about 5.5% of the population [66]. It is characterized by the presence of intermittent or persistent erythema, telangiectasia, papules, pustules on the face, and in severe long-lasting cases, phymatous lesions [66]. The pathogenesis of rosacea includes genetic factors and activation of the innate and adaptive immune response as well as neurocutaneous mechanisms [66]. Diagnosis is made based on the complete clinical picture, and treatment consists of topical agents (ivermectin, metronidazole, azelaic acid) and/or oral agents (doxycycline, lymecycline, isotretinoin). Pharmacological therapy can be enhanced by proper daily skin care and aesthetic procedures, such as the use of intense pulsed light or laser therapy [66].

Years ago, rosacea was linked to metabolic disorders, and several investigations have subsequently been published [67,68]. Rosacea has been suggested to occur more frequently in obese subjects [69], and it has conversely been suggested that patients with rosacea tend to more frequently be obese [70]. Moreover, three meta-analyses by Chen, Zhang, and Li et al. performed on large groups of patients with rosacea and controls, as well as several smaller studies, have revealed that rosacea is significantly associated with dyslipidemia [71,72,73,74], higher total cholesterol [67,71,74,75,76], LDL [67,71,74,75,76] and triglyceride concentrations [67,71]. Moreover, available studies have found no significant correlation between rosacea and HDL concentration [67,71,74]. The research group of Chen et al. tried to establish the prevalence of atherosclerosis indirectly via assessment of stroke and ischaemic heart disease, finding no relationship [71]. An inverse correlation was found by Son et al., who discovered that subjects with dyslipidemia and diabetes mellitus were more likely to have rosacea [77]. A suggested explanation for the association between rosacea and dyslipidemia is the activation of nucleotide binding oligomerization domain-like receptor 3, which can cause IL-1β release and induce structural changes of lipoproteins, decreasing their ability to break down and transport cholesterol [71]. Another theory is that cathelicidins (LL-37), which have been suggested as a key factor triggering rosacea, participate in atherosclerosis pathogenesis [72]; increased concentrations have been found in atherosclerotic plaques [78].

Two independent studies assessed epicardial fat thickness and carotid intima–media thickness volumes in patients with rosacea. Patients had significantly higher parameters compared to the control group [76,79].

The origins of the assessment of skin surface lipids in rosacea dates back to the 20th century. We are aware of only one study truly investigating skin lipidomics in subjects with rosacea. This study, by Pye et al., was performed on a very small group of patients. The authors analyzed the lipid contents in the skin, particularly cholesterol, free fatty acids, triglycerides, esters, and squalene [80]. Their study revealed no differences between patients and controls without rosacea, between males and females, or depending on the severity of the disease [80]. Another interesting study analyzing the skin barrier in rosacea revealed downregulation of the *ABCA12* gene, which is responsible for lipid transporter ABCA12, which plays an important role in lipid lamellae formation [81].

Research has shown that rosacea, despite common perception, extends beyond face-limited skin lesions. Considering these findings, several papers have recommended screening for disorders related to cardiovascular incidence to prevent increased morbidity and death [68,71,79,82]. A very engaging observation was made about the effects of oral antibiotics use in rosacea, particularly from the group of tetracyclines. In addition to their obvious antibacterial properties, they exert an anti-inflammatory influence. Tetracyclines are able to inhibit matrix metalloproteinases, which are engaged in the pathogenesis of atherosclerosis; original studies have shown that after such therapy there is a decreased expression of these enzymes in atherosclerotic carotid plaques [78]. Moreover, one study revealed that subjects treated with tetracyclines had lower risk of vascular diseases [83].

## 8. Acne Vulgaris

Acne vulgaris is probably the most common dermatosis, affecting more than 90% of the population at some point in life [84]. Its pathogenesis involves seborrhoea, improper keratinization of the pilosebaceous unit, colonization of pilosebaceous units by *Cutibacterium acnes*, and inflammation [85]. It may manifest with comedones, papules, pustules, or nodules and cysts, and the lesions are usually located on the face, back, and chest [85]. Treatment is challenging and, in addition to proper everyday care, involves topical or oral retinoids and antibiotics as well as benzoyl peroxide or azelaic acid [85].

There are several studies regarding the blood lipid profile in acne. In a study by Jiang et al. there were no differences between males and females or patients and controls in terms of total cholesterol, triglycerides, LDL, and HDL; however, after division into three subgroups based on the severity, statistically significant differences began to appear as the severity of the lesions increased. Patients had higher concentrations of total cholesterol and LDL, and in triglycerides in males. Noteworthily, the LDL-C/HDL-C ratio, which is a prognostic factor for cardiovascular complications, was significantly higher compared to controls in males with severe acne, and another marker, lipoprotein lipase, was higher in patients of both sexes [86]. A study by Yu et al. on plasma metabolomics in moderate-to-severe acne patients revealed increased concentrations of sphinganine, sphingosine, O-Phosphoethanolamine, and sphingomyelin (d18:1/18:0), which suggests a dysregulated sphingolipid metabolic pathway [87].

Many scientists have pointed to the important role of skin lipid composition in the development of acne as even more essential than increased sebum secretion itself [88,89,90]. The main source of lipids is sebocytes, followed by keratinocytes and the skin microbiome [88]. The most prevalent type of lipids in human sebum are triglycerides and fatty acids, followed by wax esters and squalene [91]. Chen et al. performed an analysis on skin surface lipids in Asian acne patients depending on age. All age groups (infants, adolescents, and adults) had increased concentration of total lipids, fatty acids, glycerophospholipids, unsaturated fatty acids, and squalene, along with decreased ceramide chain length [88]. Similar observations have been made by other scientific teams [89,90], including one study on a dark-skinned population [84]. Pappas et al. found increased levels of squalenes, wax esters and triglycerides along with reduced levels of fatty acids; however, this study was performed on a very small sample [92]. Camera et al. suggested an essential role of increased diacylglycerol concentrations in acne patients’ sebum [93]. Moreover, it seems that larger alterations in lipid composition ae associated with worse severity of acne skin lesions [88,90,93].

Chen et al. suggested that certain observed lipid alterations in acne, such as ceramide chain length and linoleic acid concentration decrease, may additionally become potential markers, along with increases in total lipid concentration, fatty acids, sterol lipids, glycerophospholipids, unsaturated fatty acids, squalene, triglycerides, ceramides, and wax esters [88]. The aforementioned changes are all associated with the pathogenesis of acne, and several of them are associated with the initiation of inflammation. Squalene, the content of which is increased after conversion to squalene peroxides, can activate lipoxygenase and be responsible for the release of proinflammatory cytokines and inflammatory condition in acne [88]. Moreover, the ratio of saturated and unsaturated fatty acids in the sebum plays an important role, as they are agonists of peroxisome proliferator-activated receptor-α (PPARα), which affects the lipid metabolism and inflammatory response. On the other hand, a decreased level of linoleic acid in the skin leads to hyperkeratosis of hair follicles, which could explain propensity to acne skin lesions [88]. In addition to age, alterations in lipid composition may be dependent on the site on the face [83], sex, psychological stress, or even circadian rhythm [89].

Interestingly, acne vulgaris has been postulated as another disease among the family of mTORC1-driven (mechanistic target of rapamycin complex 1) metabolic diseases, along with obesity, type 2 DM, and cancer [94]. Melnik suggested that the key point of acne pathogenesis in adolescence is the impact of western-type diet, which stimulates mTORC1. As it happens, a high activity is detected in acne skin lesions as well as in subjects with MS components. Therefore, young individuals with acne may be more prone to the development of MS in the future [94].

There are various subtypes of acne depending on the age of the patient (neonatal, infantile, adolescent, adult) and the type of skin lesions that patients present, each associated with slightly different pathogenesis, which may affect interpretation of the available studies.

## 9. Materials and Methods

We performed a discursive review of lipidomics in patients with selected inflammatory skin diseases. We searched the PubMed database using the following MeSH: ‘lipidomics’ and ‘lipids’ and ‘psoriasis’ or ‘atopic dermatitis’ or ‘lichen planus’ or ‘hidradenitis suppurativa’ or ‘seborrhoeic dermatitis’ or ‘acne vulgaris’ or ‘rosacea’, without date limitations. Papers in the following languages were considered: Polish, English, French, and German. The whole paper was read only if the paper indicated relevant content. Only human studies were taken into consideration; studies based only on adults or children were considered.

## 10. Discussion and Conclusions

Lipid alterations in skin diseases are apparently very frequent; hence, there is a need for further studies of lipidomics in various dermatoses which are less investigated. This knowledge would allow us to better understand the pathogenesis of these disorders and explain the dissimilarities in their variants and subtypes, especially AD, LP, and psoriasis, which could translate later into better tailored treatment. Lipidomics should be integrated with other parameters, such as genomics, proteomics, microbiomes, and exposomes. Perhaps in the future, based on reliable studies, lipidomics and other ‘omics’ might guide dermatologists in patient management. The analysis of the lipidome has been suggested as a tool to predict the progression of inflammatory skin diseases, which is a concept worthy of more attention. Unfortunately, for certain diseases the data are limited and further studies are needed to develop this branch of dermatology. In addition to patient-adjusted therapy, such research could lead to obtaining information suitable for the prognosis of disease courses in single subjects.

**Figure 2:**
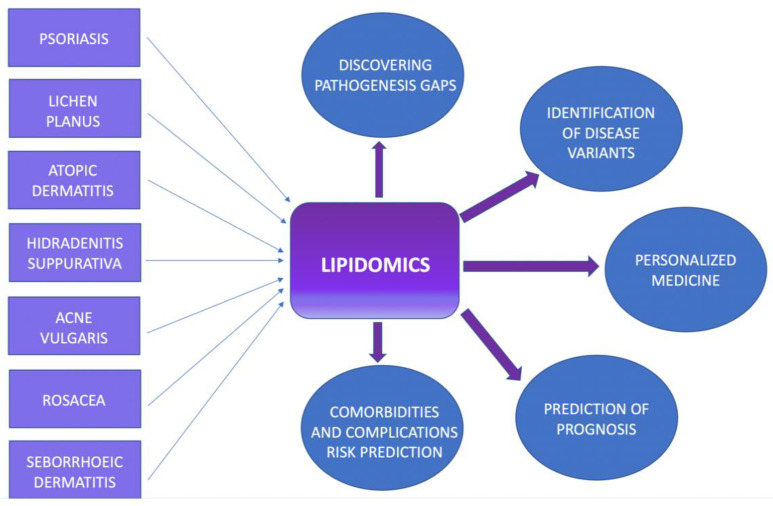
Diagram showing the benefits of analyzing lipidomics in the context of selected inflammatory skin diseases.

The other advantage of lipidomics analysis in different dermatoses is discoveries associated with lipid disturbances, which in turn translates into determining the risks that they bring. It is important to take into account that chronic inflammation in selected dermatoses plays an important role in the pathogenesis of atherosclerosis, which is a risk factor for cardiovascular complications [78]. Such a perception of the problem could lead to better management and avoidance of complications. Future studies might provide justification for screening for metabolic disorders and cardiovascular risk in subjects with other dermatoses in addition to psoriasis, and management guidelines may be formulated. A summary of the possible benefits of lipidomics analysis is presented in Figure 2.

It is worth highlighting that there have been attempts to use different biological fluids for determination of lipidomics. In addition to blood, which is moderately invasive to collect and unpleasant for patients, which may discourage them from taking part in examination, in exiting research sebum and sweat have both been used. The biggest problem seems to be skin sample collection, which is probably the most invasive biological specimen to obtain.

There are pitfalls in the interpretation of studies on this matter. First of all, we must pay attention to the included population, as well as whether it is solely adults or solely children, which may vary depending on the analyzed dermatosis; another important feature is ethnicity, which can have an impact on the course of disease; however, it is unfortunately not always clearly indicated in the descriptions of existing studies. Second, certain inflammatory skin diseases can present with various subtypes which may differ in their course. Moreover, the severity of skin lesions matters. Not every study divides patients according to the severity of lesions, and not every dermatosis can be assessed using an objective severity scale. Finally, investigation of lipidomics requires analysis of potential confounding factors such as a patient’s diet, lifestyle, and nutrition status.

## Data Availability

No new data were generated.
